# Development of an oligo DNA microarray for the European sea bass and its application to expression profiling of jaw deformity

**DOI:** 10.1186/1471-2164-11-354

**Published:** 2010-06-03

**Authors:** Serena Ferraresso, Massimo Milan, Caterina Pellizzari, Nicola Vitulo, Richard Reinhardt, Adelino VM Canario, Tomaso Patarnello, Luca Bargelloni

**Affiliations:** 1 Department of Public Health, Comparative Pathology, and Veterinary Hygiene, Faculty of Veterinary Medicine, University of Padova, Viale dell'Università 16, 35020 Legnaro, Italy; 2 Department of Evolutionary Biology, University of Florence, 50125 Florence, Italy; 3 CRIBI, University of Padova, Complesso Biologico Vallisneri, Via Ugo Bassi 58/B, Padova, Italy; 4 Max Planck Institute for Molecular Genetics, Ihnestraße 63-73, 14195 Berlin, Germany; 5 Centro de Ciências do Mar, Universidade do Algarve, Gambelas, 8005-139 Faro, Portugal

## Abstract

**Background:**

The European sea bass (*Dicentrarchus labrax*) is a marine fish of great importance for fisheries and aquaculture. Functional genomics offers the possibility to discover the molecular mechanisms underlying productive traits in farmed fish, and a step towards the application of marker assisted selection methods in this species. To this end, we report here on the development of an oligo DNA microarray for *D. labrax*.

**Results:**

A database consisting of 19,048 unique transcripts was constructed, of which 12,008 (63%) could be annotated by similarity and 4,692 received a GO functional annotation. Two non-overlapping 60mer probes were designed for each unique transcript and *in-situ *synthesized on glass slides using Agilent SurePrint™ technology. Probe design was positively completed for 19,035 target clusters; the oligo microarray was then applied to profile gene expression in mandibles and whole-heads of fish affected by prognathism, a skeletal malformation that strongly affects sea bass production. Statistical analysis identified 242 transcripts that are significantly down-regulated in deformed individuals compared to normal fish, with a significant enrichment in genes related to nervous system development and functioning. A set of genes spanning a wide dynamic range in gene expression level were selected for quantitative RT-PCR validation. Fold change correlation between microarray and qPCR data was always significant.

**Conclusions:**

The microarray platform developed for the European sea bass has a high level of flexibility, reliability, and reproducibility. Despite the well known limitations in achieving a proper functional annotation in non-model species, sufficient information was obtained to identify biological processes that are significantly enriched among differentially expressed genes. New insights were obtained on putative mechanisms involved on mandibular prognathism, suggesting that bone/nervous system development might play a role in this phenomenon.

## Background

Lower jaw protrusion or mandibular prognathism (MP) is a developmental malformation conferring a very distinctive facial phenotype. The most famous example of MP is the Habsburg family, one of the oldest European royal families, where prognathism has been observed in several successive generations [1], suggesting a strong genetic component for this disorder. In fact, although environmental factors appear to contribute to the development of MP, familiar aggregation of this character has been reported in several human populations, providing strong support to the hypothesis that heredity plays an important role in the etiology of MP. A recent study indicates the presence of a major gene that influences the expression of MP with clear signs of Mendelian inheritance (most likely autosomal dominant with incomplete penetrance), and a multifactorial component [2]. Mandibular prognathism is not limited to royal families nor to the human species, as it has been reported in several other vertebrates, *e.g. *iguanas, short-nosed dog breeds, and rabbits. In the latter species, pedigree analysis showed a simple autosomal recessive inheritance with incomplete penetrance for this condition [3]. Different types of lower jaw deformities have been reported also in several cultured as well as wild fish. In the European sea bass, *Dicentrarchus labrax*, a phenotype similar to prognatism is often observed [4]. In some cases lower jaw protrusion appears to be related to hypertrophy of the mandible (L. Bargelloni unpublished observations), in others it has been explained as an antero-posterior compression of the ethmoid region and upper jaws, with the resulting apparent protrusion of the lower jaw, and named "pugheadness". In *D. labrax*, this malformation has been attributed to a dietary excess of vitamin A as well as to absence or excess of Ω-3 poly-unsaturated fatty acids (PUFAΩ3) [5,6]. Whilst external conditions (*e.g. *diet, water temperature) are considered the most likely causative factors of bone (cranial and spinal) deformities in cultured *D. labrax*, genetics does play a role as recently demonstrated for spine malformations [7]. Likewise, genetic analysis of a population of juvenile sea bass showed a highly significant bias in the frequency of lower-jaw protrusion across different full-sib families raised under communal rearing conditions (L. Bargelloni, unpublished data).

Gene expression analysis of jaw development/deformities in early ontogenetic stages has been reported for a few candidate genes in different fish species, including the European sea bass [8,9]. Expression profiling of individuals showing alternative phenotypes has been suggested as a complementary approach to linkage analysis, in order to identify loci involved in the genetic determination of the trait (e.g. [9]). Furthermore, a transcriptomic approach might provide a broader picture of the molecular mechanisms underlying the development of cranial deformities, which could shed light also on environmental factors influencing this condition. Here, gene expression profiling of normal against jaw-deformed individuals from segregating families is reported. To this end, an oligo DNA microarray, specific for *D. labrax*, was developed, first constructing a database of unique sea bass transcripts, then annotating transcribed sequences through extensive data mining, finally designing two non-overlapping 60mer oligonucleotide probes for each transcript. Probes were synthesized *in situ *using the Agilent SurePrint™ technology to obtain a DNA microarray platform with over 40,000 probes. A similar approach has already provided robust and flexible microarray platform in other fish species [10-18]. While gene expression profiling of early developmental stages in the sea bass has been carried out using a salmonid microarray [19], to our knowledge, the present study represents the first report of a species-specific DNA microarray for *D. labrax*, either based on cDNA or oligo-DNA technology. This is quite surprising, taking into consideration the importance of the species in fisheries and aquaculture. The European sea bass is a euryhaline and eurythermic fish living in marine, estuarine, and lagoon habitats, from Scandinavia to Western Sahara in the North-eastern Atlantic Ocean and from the Western Mediterranean Sea to the Black Sea [20]. Although the sea bass supports local fisheries and recreational fishing, the main interest in this species is related to aquaculture. Sea bass hatcheries are present in several countries, from France to Greece, and sea bass farming is now widespread along the Atlantic and Mediterranean coasts from Portugal to Turkey, with a total annual production of over 134,000 metric tons (FEAP data relative to year 2008 [21]). Despite sea bass culture has greatly improved since the '80s, when the first sea bass hatcheries were established, significant bottlenecks still remain, including high frequency of cranial and skeletal malformations. These developmental abnormalities severely affect sea bass production as deformed juveniles show reduced fitness and/or marketability, requiring time-consuming and personnel-intensive manual selection to discard abnormal fish, with relevant consequences on farming costs, but also on animal welfare.

The aim of the present paper was thus to analyze mRNA expression in 38 and 58 days-old individuals, on whole heads and lower jaws respectively, using a newly developed transcriptomic platform. At the same time, expression profiling of jaw deformity represents a challenging test trial, to evaluate the performance of the platform itself.

## Results

### DLPD database

**Figure 1:**
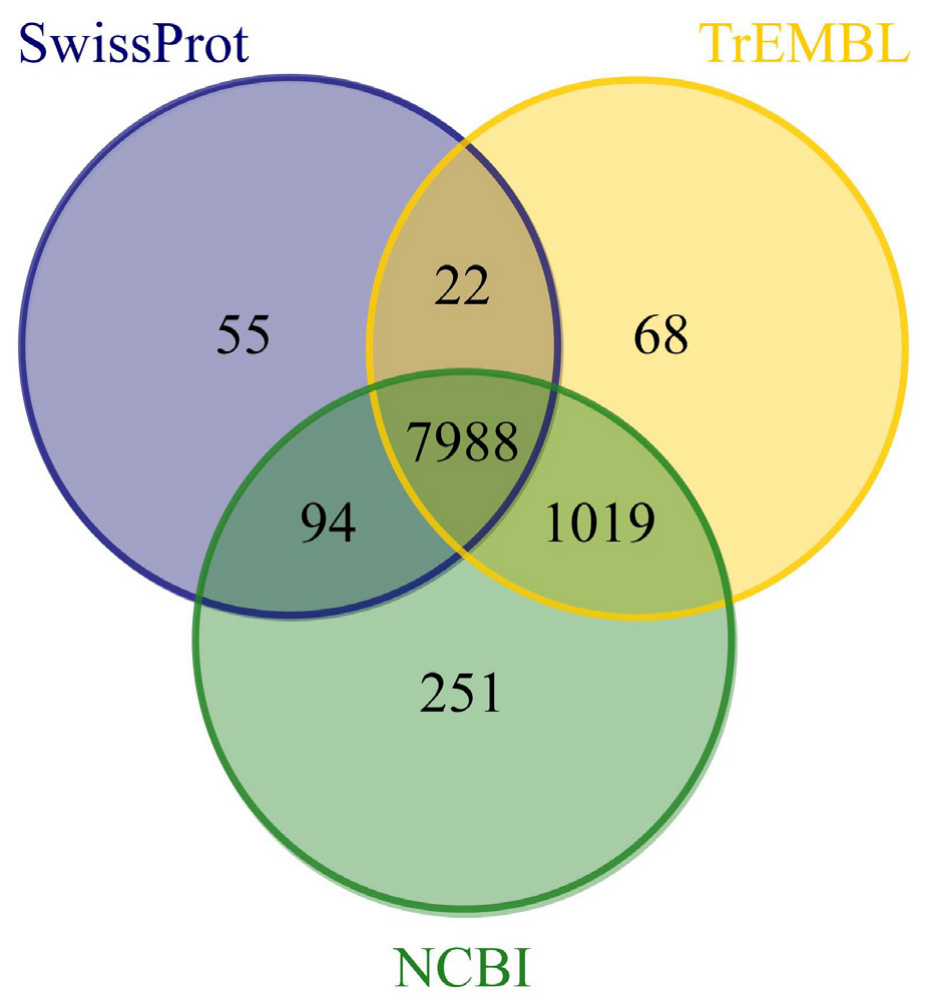
**Individual contribution of three different protein databases to the number of annotated DLPD entries**. Venn diagram showing the number of significant *matches *obtained with NCBI nr database, SwissProt and TrEMBL respectively.

A total of 19,048 unique sequences are present in the *Dicentrarchus labrax *Padova database (DLPD). Nearly half of DLPD entries (9,497) produced a significant blastx hit against either SwissProt or TrEMBL or NCBI protein databases (e-value < e^-3^). A Venn diagram showing the number of matches with each database is presented in Figure 1. Further improvement in the annotation of DLPD sequences was obtained searching the NCBI nucleotide non redundant database using the blastn option (e-value < e^-5^). This approach provided a significant match for additional 2,511 DLPD transcripts, which showed no correspondence with any known protein database, bringing the final number of DLPD entries associated with a known protein or transcript to 12,008 (63%). Fish-specific searches against five teleost transcriptomes (*Gasterosteus aculeatus, Oryzias latipes, Danio rerio, Tetraodon nigroviridis*, and *Takifugu rubripes) *identified 9,506 DLPD sequences matching against at least one species, while 4,504 ones found a significant match with all five databases (see Table 1). Additional functional information could be obtained using the Blast2GO software that allowed association of one or more GO terms to 4,692 DLPD entries. Of these, 3,267 were linked with Biological Process (BP) GO entries, 2,858 to Cellular Component (CC), and 3,879 to Molecular Function (MF). Unique GO terms represented in DLPD are 1,541 for BP, 381 for CC, and 998 for MF. A simplified view of these GO terms using a "Generic GO Slim" showed 47 BP classes, 28 for CC, and 36 for MF (see Additional File 1). All DLPD sequences are publicly available in a dedicated database (**DLPD database**: [22]), together with associated annotations, GO entries, and putative homologous genes in fish model species.

**Table 1:** Number of sea bass transcripts showing significant matches with transcriptome, publicly available on Ensembl, of five teleost species.

Ensemble transcriptomes	Species common name	Count (% of sea bass sequences)
*Gasterosteus aculeatus*	Stickleback	8448 (44.4%)

*Oryzias latipes*	Japanese medaka	7004 (36.8%)

*Takifugu rubripes*	Japanese pufferfish	7774 (40.8%)

*Tetraodon nigroviridis*	Green-spotted pufferfish	7739 (40.6%)

*Danio rerio*	Zebrafish	5579 (29.3%)

### Microarray quality assessment

Probe design was positively completed for 19,035 DLPD entries. Probe sequences and other details on the microarray platform can be found at the GEO database [23] under accession number GPL9663. Hybridization success for each probe was evaluated considering a total of 14 experiments (6 independent hybridizations for Stage 38 and 8 for Stage 58). Hybridization was considered successful when the value of flag "glsFound" was equal to 1 (see Methods). Across all experiments, only three probes (0.008%) never showed a signal higher than background, while 35,957, corresponding to 94.4% of the total number of target probes, successfully hybridized in at least half of the array experiments (see Figure S1 in Additional File 2). Six pools of Stage 38 were treated as biological replicates in order to evaluate the repeatability of array results. The degree of mutual agreement between replicates was assessed estimating Pearson correlation coefficients (r) on the entire set of expression values. Pair-wise comparisons of replicate experiments showed correlation coefficients with r > 0.99 and always significant (p-value < 0.01) (see Table 2).

**Table 2:** Correlation coefficients, on the entire set of expression values, across Stage 38 biological replicates.

	38d_N3	38d_N4	38d_N6	38d_P3	38d_P5	38d_P6
**38d_N3**	1.00					

**38d_N4**	0.996**	1.00				

**38d_N6**	0.996**	0.998**	1.00			

**38d_P3**	0.991**	0.994**	0.991**	1.00		

**38d_P5**	0.993**	0.996**	0.996**	0.994**	1.00	

**38d_P6**	0.994**	0.996**	0.997**	0.993**	0.996**	1.00

**p-value < 0.01

For each transcript two probes at non-overlapping positions as near as possible to the 3'-end of each transcript are present in the sea bass microarray platform. The variability between each two probes for the same transcript was evaluated using the ratio between their intensity levels (fold change, FC) as a measure of signal difference. This ratio is predicted to assume a value around 1 unless technical (*e.g. *poor probe quality) or biological factors (*e.g. *alternative splicing) are present, causing significant deviations from the expected. In Additional File 2 (Figure S2) each plot describes the distribution of observed fold changes between Probe_1 and Probe_2 for each array experiment in 38 days-old sea bass heads, which is symmetrical, centered around 1, and equal across all the experiments. Probe pairs show a small difference in terms of intensity values, with 68.1% of transcripts having a FC lower than 2. Pearson correlation coefficient between Probe_1 and Probe_2 for each gene across six experiments was always greater than 0.93 and highly significant (p < 0.01), further confirming the overall concordance of hybridization signal for probe pairs.

### Analysis of expression data

Raw and normalized fluorescence data of both Stage 38 and Stage 58 microarray experiments have been deposited in the GEO database [23] under accession numbers GSE19041 and GSE19001 respectively. Statistical analysis on Stage 38 whole heads showed no significant differentially expressed genes. In contrast, even after complete exclusion of hybridization data for probes with an excess of missing values (6,404 probes), analysis of filtered data showed significant differential gene expression between normal and deformed mandibles of 58 days-old juveniles. After filtering, normalized fluorescence data for 17,694 (93%) DLPD transcripts, represented by either one probe (3,722) or two probes (13,972), were subjected to a two-class SAM test. Setting FDR to 5%, a list of 333 significant probes, corresponding to 242 unique transcripts, was obtained. For 91 DLPD sequences, both Probe_1 and Probe_2 were identified by SAM. The remaining 151 transcripts were represented by a single probe. For 14 DLPD entries identified by one probe the second probe was previously excluded in the filtering step. For all the other transcripts pointed out by SAM, all second probes (except for three) were differentially expressed in prognathous individuals compared to controls (46% with a Fold-change < -2), although not significant (p-value > 0.05). All 242 transcripts were down-regulated in pooled-samples of deformed mandibles compared to controls with a FC ranging from -1.12 to -15.7. Seventy transcripts were down-regulated four-fold, with two of them more than 10-fold (see Additional File 3). A putative annotation could be obtained for 122 differentially expressed transcripts. Of the 70 most differentially-regulated transcripts (fold change < -4), half had a significant match against a known protein/DNA sequence. A substantial fraction of putative protein homologs to this "short list" of DLPD entries appears to be specific to the nervous system, including genes involved in synapsis function and neuronal development (Table 3). This observation is confirmed when analyzing the complete set of differentially regulated sea bass RNAs (*e.g.*, Neuronal membrane glycoprotein M6-a, Carboxypeptidase E, Neural Wiskott-Aldrich syndrome protein, Schwannomin-interacting protein 1, Synapsin-2B, Neuroserpin, Ephrin type-B receptor 3, Metabotropic glutamate receptor 8, RUFY3, GMP-PDE delta). Other genes with a relevant role in the development of anatomical structures can be found in the full list of differentially expressed genes (*e.g. *GH-receptor, Pleiotrophin, SOX4, Retinoic acid receptor (RXR) gamma; Additional File 3).

**Table 3:** List of the most down-regulated genes (FC ≤ -4-fold) in prognathous individuals compared to controls specific of nervous system.

Probe ID	Fold Change	Gene Description
DLPD02513_1	0.23	Synaptophysin

DLPD02650_2	0.17	Synaptosomal-associated protein 25-A (SNAP-25a)

DLPD06508_1	0.08	Synaptosomal-associated protein 25-A (SNAP-25a)

DLPD06508_2	0.08	Synaptosomal-associated protein 25-A (SNAP-25a)

DLPD03740_1	0.16	Synaptosome-associated protein

DLPD03740_2	0.17	Synaptosome-associated protein

DLPD05555_1	0.24	Vesicle-fusing ATPase

DLPD05555_2	0.25	Vesicle-fusing ATPase

DLPD07764_1	0.24	Syntaxin-binding protein 1

DLPD07764_2	0.21	Syntaxin-binding protein 1

DLPD11576_2	0.22	Neuroplastin

DLPD02151_1	0.19	Microtubule-associated protein 2

DLPD02151_2	0.16	Microtubule-associated protein 2

DLPD02425_2	0.14	Contactin-1

DLPD02075_1	0.28	Neurogenic differentiation factor 2

DLPD03006_1	0.22	Chondroitin sulfate proteoglycan 5 precursor

DLPD06176_1	0.11	Fatty acid-binding protein brain isoform

DLPD06176_2	0.11	Fatty acid-binding protein brain isoform

DLPD04679_1	0.23	Visinin-like protein 1

DLPD04899_1	0.17	Beta-synuclein

DLPD04899_2	0.22	Beta-synuclein

DLPD13372_1	0.14	Plasticin

DLPD13559_1	0.18	Secretogranin II

DLPD06571_2	0.21	Zinc finger protein of the cerebellum 3 (Zic3)

### Functional annotation of differentially expressed genes

In order to obtain a more systematic functional interpretation of the set of differentially expressed genes, GO enrichment analyses were performed following two alternative strategies. In the first one, GO enrichment analysis was performed using the GOStat tool [24]. Four GO terms with more than two gene counts were over-represented with significant uncorrected probability (see Table 4). In the second strategy, DLPD entries were linked either to human Ensembl Gene IDs or to zebrafish ZFIN IDs, following different approaches (see methods, Additional File 4), with a variable number of correspondences between sea bass and human/zebrafish sequences depending on the chosen method (see Table 5). Human or zebrafish IDs were then used in the bioinformatic tool DAVID (Database for Annotation, Visualization and Integrated Discovery) [25]. Results from direct comparison of DLPD entries against the human transcriptome yielded 60 putative human homologs of differentially expressed sea bass transcripts present in the DAVID 2008 knowledgebase. Enrichment analysis showed that 14 GO_BP terms are significantly over-represented (Table 6). Twenty six genes (43%) have a role in the regulation of biological processes (GO:0050789) and 14 are involved in the development of anatomical structures (GO:0048856). More specifically, five terms are directly related to nervous system development and function (neurological system process, synaptic transmission, regulation of neurotransmitter levels, nervous system development, transmission of nerve impulse). Two terms of GO Cellular Component (GO:0005856 "cytoskeleton", P = 0.01; GO:0019717 "synaptosome", P = 0.02; the latter one was significant, but with a low gene count), were found to be significantly over-represented. Both are related to the single entry for GO Molecular Function (GO:0008092 "cytoskeletal protein binding"), since cytoskeletal proteins are involved in synapsis genesis and function as well. Further evidence of the involvement of nervous system processes/components comes from tissue specificity of differentially regulated genes, with 39 genes showing up significantly in the brain (UP_Tissue). Similar results were obtained using PANTHER definitions (see Additional File 5).

**Table 4:** GO terms significantly over-represented, among differentially expressed genes, based on GOStat analyses

GO TERM	Biological Process	Gene Count	p-value
GO:0006813	Potassium ion transport	3	0.116

GO:0030155	Regulation of cell adhesion	2	0.116

GO:0007399	Nervous system development	4	0.116

GO:0003008	System process	4	0.116

GO:0050877	Neurological system process	3	0.116

**Table 5:** Number of DAVID identifiers retrieved with different approaches.

	ENSEMBL HUMAN GENE ID		ZFIN ID	
	
	DLPD-GA-HUMAN	DLPD-HUMAN	DLPD-GA-DR	DLPD-DR
DLPD with identifier on DAVID (Unique entries)	9,156 (6,019)	9,277 (6,458)	7,217 (4,955)	7,010 (5,148)

Identifiers uploaded as "background"	5,385	5,654	4,058	4,150

Significant DLPD with identifier on DAVID (Unique entries)	78 (75)	70 (68)	65 (63)	56 (54)

Identifiers uploaded as "gene list"	71	60	55	46

**Table 6:** GO terms significantly over-represented, among differentially expressed genes, based on DAVID analyses

	Term	Count	%	*p-value*	Fold Enrichment	FDR
**GOTERM BP_ALL**	GO:0050877 neurological system process	8	13.33%	0.006574	3.468007	11.518
	
	GO:0048856 anatomical structure development	14	23.33%	0.026081	1.852929	38.759
	
	GO:0003008 system process	9	15.00%	0.022777	2.484961	34.786
	
	GO:0032501 multicellular organismal process	23	38.33%	2.19E-04	2.116818	0.4063
	
	GO:0050789 regulation of biological process	24	40.00%	0.01166	1.561735	19.557
	
	GO:0007154 cell communication	19	31.67%	0.034946	1.558464	48.316
	
	GO:0007399 nervous system development	7	11.67%	0.039474	2.695892	52.635
	
	GO:0007275 multicellular organismal development	15	25.00%	0.011784	1.970747	19.744
	
	GO:0007268 synaptic transmission	6	10.00%	0.002618	6.04953	4.7473
	
	GO:0006811 ion transport	7	11.67%	0.022343	3.079132	34.248
	
	GO:0048731 system development	13	21.67%	0.013046	2.117152	21.624
	
	GO:0001505 regulation of neurotransmitter levels	4	6.67%	0.002972	13.29403	5.372
	
	GO:0019226 transmission of nerve impulse	6	10.00%	0.005133	5.177002	9.1072
	
	GO:0065007 biological regulation	26	43.33%	0.011622	1.508146	19.499

**GOTERM CC_ALL**	GO:0005856 cytoskeleton	10	16.67%	0.013466	2.513273	18.459

**GOTERM MF_ALL**	GO:0008092 cytoskeletal protein binding	6	10.00%	0.046655	2.96898	56.079

**UP_TISSUE**	Brain	39	65.00%	1.78E-04	1.584613	0.2548

### Natural antisense transcripts

All cDNA sequences represented in the sea bass microarray consist of public mRNA sequences or ESTs produced through 5' end sequencing of clones from directional cDNA libraries. For this reason as well as to exactly reproduce the available sea bass transcriptome without further assumptions, oligonucleotide probes were designed assuming that each unique transcript in DLPD were a "sense" strand. However, it is increasingly recognized that a substantial fraction of coding genes are transcribed on both strands, yielding sense as well as antisense transcripts. To assess whether this phenomenon occurs in the sea bass, the orientation of sea bass transcripts was compared with that of the best matching sequence in five fish species (see Methods). A conspicuous number of DLPD entries (640) showed opposite orientation against one or more putative homologs in model teleost species (see Methods). For 595 of these sea bass sequences, the matching gene(s) had always the same (opposite) orientation, while for 45 the orientation was discordant among putative homologous sequences in the matching species. A subset of DLPD entries (223) had a significant match against all five fish species, always with antisense orientation. At least, the latter likely represent natural antisense transcripts (NATs) in the sea bass transcriptome. These putative NATs appear to be generally expressed at significant levels, since 180 (80%) yielded a successful hybridization in more than half of the (14) experiments. Similar evidence was obtained for the complete set of candidate NATs (data not shown). To further explore this issue, 108 pairs of DLPD entries matching with opposite orientation a single transcript in the closest reference species, *G. aculeatus*, were identified. These sense/antisense sequences most likely represent either non-overlapping regions or alternative splicing isoforms of the same transcript, or two duplicated loci in *D. labrax *(an example is represented in Additional File 6). A comparison of hybridization signal for the corresponding probes to each pair of sense/antisense DLPD entries could therefore provide preliminary evidence on the ratio between sense and antisense transcription level at the same locus or for duplicated loci. After filtering out probe pairs with an excess of missing data (≥ 50%) across different experiments, in 38 days-old as well as 58 days-old sea bass samples sense transcripts showed higher levels of expression compared to the corresponding putative NATs, respectively in 86% (87/101) and 82% (75/91) of analyzed loci (Additional File 7). Functional annotation of the largest set of putative NATs indicated that over 50% are involved in cell metabolism, with over 20% being related to protein metabolism.

### Real-time RT-PCR validation

**Figure 2:**
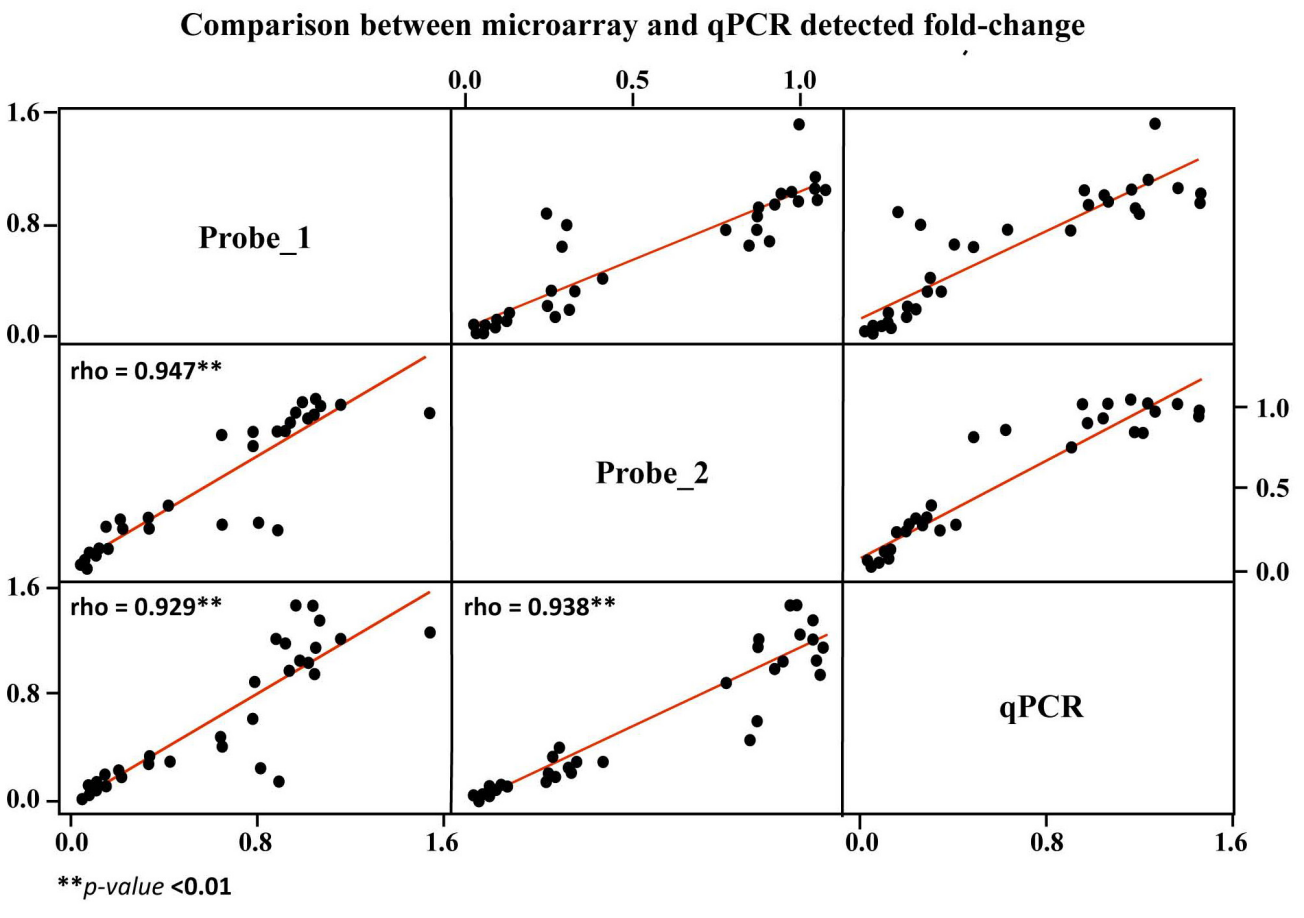
**Comparison between microarray and qPCR results**. Expression values for the 13 target genes were compared between microarray probes and Real-time RT-PCR data. In X and Y axis are reported microarray- and qPCR-estimated fold changes.

To cross-validate platform performance, a set of significant genes were tested using qRT-PCR assays. A total of 13 genes were selected for qRT-PCR analysis, within the annotated transcripts, encompassing the whole range of fold change values (1.06-13) observed in Stage 58 head samples. In order to increase the number of data points in correlation analysis between microarray and qRT-PCR results, Stage 38 samples were included in the validation procedure as well. Fold changes between Stage 38 and Stage 58 samples (an *ad hoc *quantile normalization was performed on all microarray data, 14 experiments) were calculated for 13 selected target genes. A Spearman rank correlation test was then performed considering a total of 14 experiments. A statistically significant correlation was obtained comparing expression levels for each target gene across all samples (see Table 7). Nine genes showed high correlation coefficients (Spearman rho >0.8) for both probes (p-value < 0.01) with qPCR data. Three genes exhibited a significant correlation (0.7 < rho >0.8 with p-value < 0.01) while no correlation was observed for Cyclin Dependent Kinase 2-associated protein 1 (CDK2) exhibiting a fold change < 1.2. Fold changes detected by gene-specific PCR assay and by both microarray probes (1 and 2) for the same target transcript were also compared. Fold change was calculated as the ratio of mean signal intensity between prognathous and controls on both Stage 38 and Stage 58 as well as between Stage 38 and Stage 58. For all tested targets, the direction of change in expression was concordant between qPCR and microarray results. For both probes, linear regression of microarray-estimated fold change against qPCR results demonstrated a strong positive correlation (Spearman rho >0.92, p-value < 0.01) between the two technologies (see Figure 2).

**Table 7:** Correlation between microarray and Real-time RT-PCR expression data.

DLPD ID	Gene Name	Spearman's rho		
		
		Probe_1/Probe_2	qPCR/Probe_1	qPCR/Probe_2
**DLPD03006**	Chondroitin sulfate proteoglycan 5 Precursor	0.867**	0.878**	0.912**

**DLPD08826**	Calcitonin-related peptide	0.887**	0.796**	0.846**

**DLPD03840**	Stathmin	0.978**	0.930**	0.974**

**DLPD11717**	Tetraspanin	0.991**	0.758**	0.734**

**DLPD13372**	Peripherin	0.979**	0.951**	0.895**

**DLPD06176**	Brain specific fatty acid protein	0.978**	0.960**	0.974**

**DLPD02075**	Neurogenic differentiation factor	0.863**	0.830**	0.907**

**DLPD11576**	Stromal cell derived receptor 1β	0.958**	0.762**	0.846**

**DLPD03024**	CDK2-associated protein 1	0.952**	0.162	0.086

**DLPD06508**	Synaptosomal associated protein	0.986**	0.902**	0.874**

**DLPD12789**	Pleiotropin	0.979**	0.958**	0.944**

**DLPD07155**	GRAM-domain containing protein 2 (splicing variant 1)	0.900**	0.934**	0.855**

**DLPD09843**	GRAM-domain containing protein 2 (splicing variant 2)	0.991**	0.855**	0.890**

** p < 0.01

## Discussion

Four major points emerge from the results obtained in the present study. The first one regards the high level of flexibility, reliability, and reproducibility of the microarray platform developed for the European sea bass. Oligonucleotide probe design proved to be feasible on nearly all available transcribed sequences and almost every designed probe yielded a successful hybridization signal, providing a most complete representation of the current transcriptome for *D.labrax*. One of the most important requirements for a microarray platform is good reproducibility. Analysis of biological replicates showed extremely high correlation coefficients, demonstrating great reproducibility of microarray data. As already reported [16,18], the use of a one color labeling scheme has apparently no negative effects on the quality of data, but it greatly simplifies the experimental design and allows easier analysis of novel samples. Finally, validation with qRT-PCR, a method that is based on a different technical approach, confirmed microarray hybridization data with the exception of a single gene, CDK2-associated protein 1, which showed a positive, but not significant correlation between results of the two different methodologies (Table 7). This is likely due to the small difference in expression for this gene (mean fold change estimated from array data is 1.3). Lack of correlation between microarray and qRT-PCR for genes exhibiting low levels of change (< 1.4 fold) has been commonly reported. Indeed a two-fold change is usually considered as the cut-off below which microarray and qRT-PCR data begin to lose correlation [26].

The second point concerns the annotation of the sea bass transcriptome. Whilst the design and validation of a custom oligo DNA microarray has become a straightforward procedure, a proper functional classification for the collection of unique transcripts that are represented onto the DNA microarray is certainly more difficult to achieve in non-model species, particularly in teleost fish.

High-throughput technologies have now prompted different comprehensive approaches toward functional analysis of lists of differentially expressed genes/proteins, Gene Ontology [27] being the most popular of them. These methods offer relevant advantages, although gene ontology annotations suffer from known limitations [28]. In non-model species these approaches are based on putative homology against better characterized organisms rather than on direct experimental evidence. There are here two major sources of error; the first related to the problem of identifying true orthology [29], the second to the fact that sequence homology does not always imply functional homology, due to inherent differences between the species compared. In the case of teleost fish, extracting gene functional annotations from mammalian models poses an additional problem. The ancestral teleost genome underwent a whole genome duplication (WGD) after the separation from the tetrapod lineage [30], which led to the presence of two copies of each gene present in higher vertebrates, a fraction of which genes was retained through different mechanisms (*e.g. *neofunctionalization, subfunctionalization, genomic inertia), whereas others have been lost by gene deletion or pseudogenization [31]. A ready example of such phenomenon is provided by the gene that showed the largest fold change difference in the present study, Arrestin 3 (Retinal X arrestin, ARR3) (see Additional file 3). ARR3 belongs to the cluster of beta-arrestins, which includes four paralogs (Arrestin beta 1, ARRB1; Arrestin beta 2, ARRB2, S-AG arrestin, ARR3) in the human genome [32] but at least seven paralogs (ARRB1a; ARRB1b, ARRB2a, ARRB2b, S-AG arrestin, ARR3a, ARR3b) in the stickleback genome. Despite all these limitations, functional annotation through sequence homology remains the best available method in non-model species. In the case of *D. labrax*, a good percentage (>60%) of DLPD entries has a significant match with a known gene/protein, generally higher than the value observed in similar studies on other species (gilthead sea bream 40% [16], turbot 50.7% [33] Senegal sole 40.6% [34], largemouth bass 46% [10], pre-smolt Atlantic salmon 50.3% [35], channel catfish 51% [36], and comparable to that reported for the Atlantic halibut 60% [37]). A smaller proportion (>4,500) of sea bass transcripts could be associated with a GO term, most likely as a result of the more stringent criteria enforced in Blast2GO and the lack of GO annotation for part of the protein sequences matching DLPD entries. Nevertheless, sufficient information was obtained to construct a custom background, which takes into account the fact that the sea bass transcriptome is only partly represented onto the microarray, and to identify biological processes that are significantly enriched among differentially expressed genes (see Table 4). A second approach of annotation by similarity, through direct or indirect comparison against a model species transcriptome appears to convey comparable information, with over 6,000 human or 5,000 zebrafish putative homologs. The use of the human transcriptome as a reference provides a larger number of significant functional annotations (see Table 5), which is somehow expected since the probability of identifying significantly enriched GO terms depends in part on the size of the gene list to be analyzed and the corresponding background. On the other hand, using human-centered annotations might be more prone to the risks described previously. Finally, the results obtained with different methods (GOStat compared to DAVID, DAVID human knowledgebase against zebrafish one) show a certain degree of overlap, yet there are terms that appear only in one or two analyses, therefore the obtained results should be interpreted with some caution.

While the majority of DLPD entries can be associated to a known gene, a substantial "silent" minority exists of transcripts that could not find any significant match against a variety of sequence databases. Even if not associated with a known coding gene, these transcripts might convey useful information. For instance, a large proportion (190/242 with a threshold of e^-5^, and 169/242 with a threshold of e^-10^) of all differentially expressed transcripts in jaw deformed sea bass find a significant match against a specific region in the stickleback genome. The strong conservation of large genome segments between *D. labrax *and *G. aculeatus *(R. Reinhardt, unpublished data) and the availability of a physical map of the sea bass genome (F. Galibert, personal communication) will allow the use of matching DLPD transcripts as positional candidates to identify loci involved in the genetic determination of mandibular prognathism.

The 40% of non-matching DLPDs likely represents different types of transcripts, *e.g. *5' and 3' end untranslated regions (UTRs) or alternative splicing isoforms that have not been (yet) characterized in other species, extremely fast-evolving protein-coding regions, novel genes. In addition, a fraction of non-matching sea bass transcripts might belong to the expanding universe of non-coding RNAs, which appear to cover an increasing part of the animal genome [38,39]. Although originally dismissed as transcriptional noise, evidence is accumulating for a functional role of these transcripts [40]. The third point of the present study, the presence of NATs in the sea bass transcriptome, is related to this issue. Natural antisense transcripts have been originally identified searching EST collections, and appear to be widespread across animal species, albeit at diverse frequency [41]. Various putative functions have been proposed for NATs [42], with an increasingly relevant role in the production of endogenous siRNAs [43]. Oligo DNA microarrays have been used to specifically detect sense-antisense gene expression [44,45], although there are some caveats about the risk of false positive NAT detection due to genomic DNA contamination of RNA extracts or unintended labeling of both cDNA strands [46]. Microarray analysis of the sea bass transcriptome was not specifically aimed at investigating NATs yet it provided preliminary evidence for the existence of putative antisense RNAs in this species. Based on sequence homology with the stickleback genome, in most cases sea bass NATs represent non-overlapping regions of corresponding sense transcripts (see Additional File 6), and can derive from protein-coding regions as well as non-protein-coding sequences, including exonic, intronic and intergenic sequences. While the biological roles of NATs remain to be elucidated, putative sea bass NATs show lower levels of expression compared to the corresponding sense DLPD transcripts (see Additional File 7), as already reported for other species [46]. However, more than 10% of putative NATs showed higher expression levels compared to sense transcripts (*e.g. *14 in 38 days-old larvae with fold change ranging between 1.4 and 71). Functional analysis of sea bass NATs also showed significant enrichment of certain molecular functions, although these results require further confirmation.

The fourth and last evidence from the present study was the discovery of a set of differentially regulated transcripts in the mandible of jaw-deformed juvenile sea bass compared to normally developed animals. Developmental defects are generally thought to trace back early in the ontogeny. For instance, mutations in master regulatory genes start to exert their effects during early patterning of craniofacial development (*e.g. *Tbx22 in mammals and zebrafish [47], Endothelin1 in several vertebrates [48]), while precocious treatment of Japanese flounder larvae (6-9 days post-hatching) with retinoid acid receptor agonists produces lower jaw deformities [49]. In the present study, transcriptional changes are observed at a much later stage, which might represent either the downstream effects of earlier changes in the expression of hierarchically higher genes and/or transcriptional perturbations that start at a less differentiated stage and are maintained until later stages. On the other hand, no statistically significant differences in the expression profile of 38-days old sea bass juveniles were observed comparing deformed against normal fish. For this stage, however, whole heads were analyzed, which might have caused a reduced sensitivity in detecting differential expression. Using image analysis on juvenile sea bass pictures it was estimated that the lower jaw represents less than 1/10 of the entire head, while the anterior region of the mandible, the dentary, is approximately 1/30 of the sampled tissues. This means that if for instance a certain gene has an average expression level of 10 in the whole head whereas it is up-regulated 10-fold (100) exclusively in the mandibular region of deformed animals, the observed overall fold change would be 1.9, and in case differential expression is limited to the dentary a 1.3 fold increase would be present, likely below the threshold of reliable detection using microarray analysis. Microdissection of the mandible or part of it will therefore be required to identify differentially expressed genes in deformed animals at early stages, as reported here for 58-days old juveniles. The relatively large set of down-regulated transcripts in the lower jaw of prognathous fish also suggests that mandibular prognathism observed in the present study might be distinct from pugheadness, which is reported to be the result of under-development of the upper jaw, with only apparent protrusion of the normally developed lower jaw. As in most teleost fish, mandible development in *D. labrax *is characterized by the presence of a cartilagenous component, the Meckel's cartilage, which is established early and subsequently regresses and ossifies, and a dermal bone component, the dentary, which appears later (25 days post hatching) and undergoes direct (membranous) ossification [50]. Both components receive an important contribution of cells originating from the neural crests [51]. The ossification process of the Meckel's cartilage is relatively well known, with a central role of the Hedgehog pathway, and it appears to be conserved in fish and mammals [52], whereas the molecular mechanisms controlling the dentary development are less characterized and seem to be at least partially distinct from those observed in the Meckel's cartilage [53]. How can this relate to the genes that were found to be differentially expressed in the present study? A significant enrichment in regulatory genes, especially in the development of anatomical structures was obtained in GO functional annotation, and gene-by-gene evaluation confirmed that some of them might be linked to bone formation. For instance, SOX4 has been reported to act downstream to Parathyroid Hormome (PTH) and PTH related protein (PTHrP) in osteoblast-like cells, being highly expressed in hypertrophic condrocytes during the mineralizing phase of endochondral ossification [54]. Pleiotrophin or HB-GAM is highly expressed in bone, where it seems to play a role in bone development and remodelling [55]. Retinoic acid receptor X gamma (RXRγ) forms heterodimers with vitamin D receptors, Peroxisome prolifireator-activated receptors (PPARs), and Retinoic acid receptors (RARs). All these nuclear receptors are somehow involved in bone formation and more in general in controlling skeletal growth. For instance, vitamin A and its metabolites, acting through RARs, have been reported to cause vertebral and craniofacial deformities in farmed fish [5]. Remarkably, high levels of dietary vitamin A significantly increased the frequency of cranial malformations (underdeveloped lower jaw) in *D. labrax *[5] as well as in other fish species [49,56].

It seems, however, that bone development is not the most represented biological process when examining the functional roles of protein encoded by differentially expressed genes in deformed sea bass. A substantial fraction of them appears to affect nerve growth and function. This evidence might be explained by a temporal shift in the development of mandibular nerves, which in turn may be a consequence of the altered process of mandible bone formation. Down-regulation of several markers of neuronal development seems to suggest a delay in the differentiation of neuronal cells that constitute the nervous component of the lower jaw. However, there could be a closer relationship between mandibular bone formation and transcriptional changes for neuron-specific genes. Increasing evidence indicates that the nervous system participates in the regulation of bone physiology [57]. Peripherally-released neurotransmitters exert their actions on osteoblasts and osteoclasts, which have been demonstrated to express specific receptors for these mediators. Of particular interest for the present study is the role of calcitonin gene-related peptide (CGRP), which is a neuropeptide with a well-established function in bone metabolism, promoting bone formation and repressing bone resorption [58]. As other neuromediators, CGRP is also produced directly by osteoblasts as an autocrine factor [57]. The expression of neuronal-specific genes in bone cells is not limited to neurotransmitters and their receptors, but it extends to the molecular network for regulated glutamate exocytosis (*e.g. *SNARE, SNAP-25, syntaxin, synaptophysin, syntaghmin) generally described in pre-synaptic nerve terminals [59]. The role of glutamate signalling in osteoblasts and osteoclasts is complex and *in vivo *studies are still limited [60]. Evidence from conditional KO mice lacking components of the glutamate pathways showed reduced mineralization and delayed ossification. Several genes involved in exocytosis and glutamate signalling are represented among down-regulated transcripts in the present study (*e.g. *SNAP-25, Synaptophysin, Synapsin, Metabotropic glutamate receptor 8). A working hypothesis to hold together the above evidence might be that in jaw-protruding sea bass the bone formation process is delayed compared to normally developed animals, through down-regulation of different signalling pathways, which control bone formation/remodelling, either directly in osteoblasts and/or in neuronal terminals innervating the mandible. A delay in the ossification process might allow a prolonged growth of the mandibular bone components resulting in a protruding lower jaw.

Finally, it should be noted that part of the transcriptional differences observed in deformed animals might point, at least in part, to other regions of the dissected mandible. For instance, the formation of the tongue and/or the teeth might be indirectly affected by the deformity, and contribute to the differential gene profile. This in turn might provide a complementary/alternative hypothesis to explain the prevalence of neuron-related transcripts. In fact, tooth development has been shown to be tightly linked with nerve development.

Clearly, the above hypotheses await further confirmation from additional developmental stages and with the use of methods that allow better dissection of anatomical structures.

## Conclusion

In conclusion, assembly and annotation of a first version of the European sea bass transcriptome led to the construction and validation of a species-specific oligonucleotide microarray. Microarray analysis of the sea bass transcriptome provided preliminary evidence for the existence of putative antisense RNAs in this species. This genomic platform was applied to detect differentially expressed genes in the mandible of jaw-deformed fish, revealing significant down-regulation of several transcripts involved in bone formation and neuronal function.

## Methods

### Sample collection and RNA extraction

Two developmental stages, 38 days-old (Stage 38, average length 12 mm) and 58 days-old (Stage 58, average length 16 mm) sea bass juveniles, were included on the experimental design. The animals were collected at the fish farm "Impianto di Acquacoltura Ca' Zuliani" (Pila di Porto Tolle, Italy), not subjected to any experimental manipulation and sacrificed, using an excess of anesthetic, according to guidelines of the Italian law (DL 116/92) and the European legislation (Council Directive 86/609/EEC and subsequent amendments). For Stage 38, the cranial region was dissected under a stereomicroscope for 15 normal and 15 jaw-deformed individuals. For Stage 58, it was possible to dissect the lower jaw for a total of 40 individuals (20 normal fish and 20 deformed ones). Independent tissue pools (3 for Stage 38 and 4 for Stage 58) of five fish each were produced for each condition/stage combination.

Total RNA was extracted from pooled tissue samples using the RNAeasy Mini Kit (Qiagen, Hilden, Germany) following the manufacturer's instructions. RNA quality was previously checked by gel electrophoresis on a 1% agarose gel containing SYBR Safe™ DNA Gel stain 10,000× (Invitrogen™, Carlsbad, California). RNA concentration was determined using a UV-Vis spectrophotometer, NanoDrop^® ^ND-1000 (NanoDrop Technologies, Wilmington, USA). RNA integrity and quality was finally estimated on an Agilent 2100 Bioanalyzer (Agilent Technologies, Palo Alto, CA). RNA Integrity Number (RIN) index was calculated for each sample using Agilent 2100 Expert software. RIN provides a numerical assessment of the integrity of RNA that facilitates the standardization of the quality interpretation. In order to reduce experimental biases in microarray analysis due to poor RNA quality, only RNA samples with RIN number >8 were further processed.

### Database construction, annotation, and probe design

Unique sequences were assembled starting from an existing assembly (version 1.0 Adl1) that could be downloaded from the SIGENAE web site [61] at the starting time of the present study (February 2008), which originated from 14 normalized cDNA libraries representing different adult tissues (liver, ovary, testis, bone and cartilage, heart and vessel, brain and pituitary gland, adipose tissue, head kidney, trunk kidney, gill, spleen, intestine, muscle, skin), one larval cDNA library, and two subtracted libraries from sea bass challenges in freshwater and seawater. This initial set of 17,623 assembled unique sequences was clustered with 5,045 ESTs from a normalized cDNA library of the corpuscle of Stannius, 2,356 publicly available ESTs in GenBank, and 226 public full-length or partial mRNA sequences. Clustering was carried out using a strategy based on BLAST (Basic Local Alignment Search Tool) to identify candidate sequences (cut off e-value set to e-10) to be included in a cluster and Cap3 [62] to perform the assembly and produce the consensus sequences. ESTs were considered to belong to the same cluster if there was an overlap of at least 40 bp and an overlap identity of 90%. The clustering pipe-line produced a final set of 19,734 different clusters. Links between GenBank accession numbers and unique transcripts in the database presented here are reported in the GEO "Platform data table" (GPL9663).

Similarity searches for each unique transcript against different databases were performed using the BLAST. The procedure involved two different steps: i) BLAST search with blastx option (cut off e-value of < 1.0 e-3) against the NCBI (National Centre for Biotechnology Information) amino acid non-redundant database, SwissProt database, and TrEMBL database ii) BLAST search with blastn option (cut off e-value of < 1.0 e-5) against NCBI nucleic non-redundant database and Ensembl transcriptomes for the five fish species which have a high quality draft genome sequence zebrafish (*Danio rerio*), stickleback (*Gasterosteus aculeatus*), Japanese medaka (*Oryzias latipes*), Japanese pufferfish (*Takifugu rubripes*), and green-spotted pufferfish (*Tetraodon nigroviridis*)). Gene ontology (GO) associations for "Biological process", "Molecular function" and "Cellular component" were obtained using the blastx option against the NCBI amino acid non redundant database as implemented in the Blast2GO software [63]. A summary of the overall results of GO annotation was obtained with the CateGOrizer program [64], employing the "Generic GO slim" set [65]. All unique sea bass transcripts, and their corresponding annotations are stored in the *Dicentrarchus labrax *Padova Database (DLPD), which is based on the BIOMART environment and can be queried using different filters based on contig name, description, GO terms, or for a combination of these criteria. It is possible to visualize different attributes choosing among the sequence name, the sequence contig consensus, sequence description, GO annotation, and putative orthologous genes in the human genome as well as in five fish model species (*G. aculeatus, O. latipes, D. rerio, T. nigroviridis*, and *T. rubripes*).

Two non-overlapping probes for each unique transcript were designed to construct a high-density oligo-DNA microarray. Probe design was carried out using the Agilent eArray interface [66], which applies proprietary prediction algorithms to design 60mer oligo-probes. Microarrays were synthesized in situ using Agilent non-contact ink-jet technology with a 4 × 44K format. Each array included default positive and negative controls.

### RNA labeling and hybridization

Sample labeling and hybridization were performed according to the Agilent One-Color Microarray-Based Gene Expression Analysis protocol. Briefly, for each sample 500 ng of total RNA were linearly amplified and labeled with Cy3-dCTP. A mixture of 10 different viral poly-adenilated RNAs (Agilent Spike-In Mix) was added to each RNA sample before amplification and labeling, to monitor microarray analysis work-flow. Labeled cRNA was purified with Qiagen RNAeasy Mini Kit, and sample concentration and specific activity (pmol Cy3/μg cRNA) were measured in a NanoDrop^® ^ND-1000 spectrophotometer. A total of 1,650 ng of labeled cRNA was prepared for fragmentation adding 11 μl 10× Blocking Agent and 2.2 μl of 25× Fragmentation Buffer, heated at 60°C for 30 min, and finally diluted by addition with 55 μl 2× GE Hybridization buffer. A volume of 100 μl of hybridization solution was then dispensed in the gasket slide and assembled to the microarray slide (each slide containing four arrays). Slides were incubated for 17 h at 65°C in an Agilent Hybridization Oven, subsequently removed from the hybridization chamber, quickly submerged in GE Wash Buffer 1 to disassembly the slides and then washed in GE Wash Buffer 1 for approximately 1 minute followed by one additional wash in pre-warmed (37°C) GE Wash Buffer 2.

### Data acquisition and analysis

Hybridized slides were scanned at 5 μm resolution using an Agilent G2565BA DNA microarray scanner. Default settings were modified to scan the same slide twice at two different sensitivity levels (XDR Hi 100% and XDR Lo 10%). The two linked images generated were analyzed together and data were extracted and background subtracted using the standard procedures contained in the Agilent Feature Extraction (FE) Software version 9.5.1. The software returns a series of spot quality measures in order to evaluate the goodness and the reliability of spot intensity estimates. All control features (positive, negative, etc.) except Spike-in (Spike-In Viral RNAs) were excluded from subsequent analyses. Spike-in control intensities were used to identify the best normalization procedure for each dataset. After normalization, spike intensities are expected to be uniform across the experiments of a given dataset. Normalization procedures were performed using R statistical software [67]. On our data quantile normalization always outperformed cyclic lowess and quantile-normalized data were used in all subsequent analyses.

Statistical tests implemented in the program Significance Analysis of Microarray (SAM) [68] were used to identify differentially expressed genes between normal and deformed animals for both stages. For Stage 58 analysis, probes with a high proportion of missing values (more than two missing values across the biological replicates of each condition) were removed from the dataset. The flag "glsFound" (set to 1 if the spot has an intensity value significantly different from the local background, 0 otherwise), obtained after data processing with Feature Extraction Software 9.5.1, was used to identify unreliable spots, probes with flag equal to 0 were considered as "missing".

Pearson correlation coefficients were estimated within and among arrays with Statgraphics Centurion XVI to evaluate repeatability and precision of the obtained microarray data. A non parametric Spearman rank-correlation test was used to assess correlation between expression values measured respectively with real-time RT-PCR and microarray. The same test was performed separately for each microarray probe. Spearman correlation tests were implemented using SPSS 12.0.

### Functional annotation

Functional annotation of differentially expressed genes was performed using the DAVID web-server [69] and GOstat [70]. .For GOStat analysis, all significantly down-regulated transcripts with at least one associated GO description (39), previously obtained with Blast2GO, were compared against a background of 4,692 GO-annotated DLPD entries, using the GOStat program with default settings. Since DAVID databases contain functional annotation data for a limited number of species, it was necessary to link DLPD transcripts with sequence identifiers that could be recognized in DAVID (Ensembl Human Gene IDs and ZFIN-IDs). This was carried out through dedicated BLAST searches implemented as follows: i) blastx and blastn BLAST options were both used to search significant matches of DLPD sequences directly against human Ensembl proteins and transcripts respectively ii) the same search strategy was implemented first against Ensembl proteins and transcripts of *G. aculeatus*, and then using best-hits either from blastx (stickleback proteins) or blastn (predicted proteins from stickleback transcripts) search as queries in a second search, with blastp option, against all Ensembl human proteins, iii) a direct search using either blastn or blastx against all zebrafish Ensembl proteins, iv) an indirect search first through the stickleback protein and transcript databases in Ensembl and then recovering zebrafish putative orthologous proteins as described above for human proteins (ii). Finally, Ensembl Human Gene IDs and ZFIN-IDs were obtained from the corresponding Ensembl protein entries using the BIOMART data mining tool [71] A scheme summarizing the four different approaches is presented in Additional File 4. Human or zebrafish IDs genes corresponding to differentially expressed sea bass transcripts and to all genes represented on the array were then used to define respectively a "gene list" and a "background" in the bioinformatic tool DAVID. DAVID was then used with the following settings, gene count = 4 and ease = 0.05

### Validation of gene expression data using quantitative (q) RT-PCR

Thirteen target genes and one reference gene (Malate dehydrogenase, MDH) were selected for qRT-PCR analysis. Gene-specific primers were defined for each transcritpt with the Primer 3 v.0.4.0 software. To design intron-spanning assays, intron-exon boundaries were identified through the alignment, whenever possible, with the corresponding genomic sequences available from the European sea bass genome sequencing project (R. Reinhardt, unpublished data). Otherwise, putative intron-exon junctions were inferred by comparison with homologous genes present in high-quality draft fish genomes.

One microgram of total RNA for each sample was reverse transcribed to cDNA using Superscript II (Invitrogen™, Carlsbad, California). An aliquot (2.5 μl) of diluted (2 ng/μl) cDNA template was amplified in a final volume of 10 μl, containing 5 μl Platinum SYBR Green qPCR SuperMix-UDG 2× (Invitrogen™) and 0.25 μl of each gene-specific primer (10 μM). The amplification protocol consisted of an initial step of 2 min at 50°C and 2 min at 95°C, followed by 45 cycles of 10 s at 95°C and 30 s at 60°C. All experiments were carried out in a LightCycler^® ^480 (Roche Diagnostics, Mannheim, Germany). To evaluate the efficiency of each assay, standard curves were constructed amplifying two-fold serial dilutions of the same cDNA (38d_N3), which was used as calibrator. For each sample, the Cp (Crossing point) was used to determine the relative amount of target gene; each measurement was made in duplicate, and normalized to the reference gene (MDH, contig name DLPD06340), which was also measured in duplicate. MDH was chosen as reference gene in qRT-PCR assays as it is considered a housekeeping gene, and it did not exhibit any significant change in microarray data between either the two developmental stages or the two conditions (normal and deformed) tested (percentage coefficient of variation for Probe_1 and Probe_2 was 13.4% and 13.5% respectively). Samples tested in qRT-PCR were the same of microarray experiments. One biological replicate of Stage 38 (38d_N3) was used as calibrator, the internal control for each amplification reaction.

## Authors' contributions

LB, TP, and AVMC conceived and designed the project. RR produced the EST sequences. NV and CP conceived and constructed the database. MM and SF carried out probe design and editing, performed microarray experiments and validated array data with qRT-PCR. SF executed all statistical analyses. MM performed functional annotation analyses. LB, SF and MM wrote the manuscript. All listed authors edited the manuscript. All authors read and approved the manuscript.

## Supplementary Material

Additional file 1 **GO terms associated to DLPD represented in D.labrax microarray using "Generic GO slim" in Blast2GO software**. Details about "Biological process", "Molecular function" and "Cellular component" GO terms. Click here for file

Additional file 2 **Microarray quality assessment**. Hybridization success across all experiments (Figure S1) and distribution of observed fold changes between Probe_1 and Probe_2 (Figure S2) are reported. Click here for file

Additional file 3 **List of significant probes identified by SAM analysis**. Down-regulated genes in pooled samples of deformed mandibles compared to controls. For each transcript, fold change, q-value, description and GO terms are reported. Click here for file

Additional file 4 **Scheme illustrating the four different approaches tested for retrieving DAVID identifiers**. DLPD entries were linked either to human Ensembl Gene IDs or to zebrafish ZFIN IDs directly or passing through stickleback sequences. Click here for file

Additional file 5 **PANTHER terms significantly represented among differentially expressed genes**. Results obtained using DAVID (Database for Annotation, Visualization, and Integrated Discovery) 2008. Click here for file

Additional file 6 **Examples of DLPD entries matching, on opposite orientation, the same stickleback transcripts**. **A**. DLPD03928 shares the same orientation with ENGACT000000021382 and shows higher levels of expression compared to the corresponding antisense transcript (DLPD06020). The opposite situation is represented in **B**. DLPD01599 has opposite orientation to its putative stickleback homologue (ENGACT000000021382) and is over-expressed compared to the corresponding sense transcripts (DLPD10744). Blast E-value, microarray fluorescence signal for each DLPD entry, and fold change between sense and antisense DLPD transcripts are also reported. Click here for file

Additional file 7 **Fold changes of sense transcripts compared to the corresponding putative NATs in 38 and 58 days-old sea bass samples**. Click here for file
