# Supplementary material for: Development of an oligo DNA microarray for the European sea bass and its application to expression profiling of jaw deformity
Source: BMC Genomics. 2010 Jun 3;11:354. doi: 10.1186/1471-2164-11-354 (PMC2889902; doi:10.1186/1471-2164-11-354)
Supplement: Additional file 1 — GO terms associated to DLPD represented in D.labrax microarray using "Generic GO slim" in Blast2GO software. Details about "Biological process", "Molecular function" and "Cellular component" GO terms. [file 1471-2164-11-354-S1.DOC]

**BIOLOGICAL PROCESS GO terms**

| **GO:0008150 Biological Process** | | | |
| --- | --- | --- | --- |
| **GO class ID** | **Definition** | **Count** | **Fraction** |
| [GO:0008152](http://amigo.geneontology.org/cgi-bin/amigo/go.cgi?action=query&view=details&search_constraint=terms&query=GO:0008152) | metabolism | 2749 | 21.82% |
| [GO:0019538](http://amigo.geneontology.org/cgi-bin/amigo/go.cgi?action=query&view=details&search_constraint=terms&query=GO:0019538) | protein metabolism | 992 | 7.87% |
| [GO:0009058](http://amigo.geneontology.org/cgi-bin/amigo/go.cgi?action=query&view=details&search_constraint=terms&query=GO:0009058) | biosynthesis | 857 | 6.80% |
| [GO:0006139](http://amigo.geneontology.org/cgi-bin/amigo/go.cgi?action=query&view=details&search_constraint=terms&query=GO:0006139) | nucleobase, nucleoside, nucleotide and nucleic acid metabolism | 800 | 6.35% |
| [GO:0006810](http://amigo.geneontology.org/cgi-bin/amigo/go.cgi?action=query&view=details&search_constraint=terms&query=GO:0006810) | transport | 738 | 5.86% |
| [GO:0007275](http://amigo.geneontology.org/cgi-bin/amigo/go.cgi?action=query&view=details&search_constraint=terms&query=GO:0007275) | development | 689 | 5.47% |
| [GO:0007154](http://amigo.geneontology.org/cgi-bin/amigo/go.cgi?action=query&view=details&search_constraint=terms&query=GO:0007154) | cell communication | 536 | 4.25% |
| [GO:0007165](http://amigo.geneontology.org/cgi-bin/amigo/go.cgi?action=query&view=details&search_constraint=terms&query=GO:0007165) | signal transduction | 468 | 3.71% |
| [GO:0016043](http://amigo.geneontology.org/cgi-bin/amigo/go.cgi?action=query&view=details&search_constraint=terms&query=GO:0016043) | cell organization and biogenesis | 433 | 3.44% |
| [GO:0006464](http://amigo.geneontology.org/cgi-bin/amigo/go.cgi?action=query&view=details&search_constraint=terms&query=GO:0006464) | protein modification | 375 | 2.98% |
| [GO:0006350](http://amigo.geneontology.org/cgi-bin/amigo/go.cgi?action=query&view=details&search_constraint=terms&query=GO:0006350) | transcription | 368 | 2.92% |
| [GO:0009056](http://amigo.geneontology.org/cgi-bin/amigo/go.cgi?action=query&view=details&search_constraint=terms&query=GO:0009056) | catabolism | 307 | 2.44% |
| [GO:0009653](http://amigo.geneontology.org/cgi-bin/amigo/go.cgi?action=query&view=details&search_constraint=terms&query=GO:0009653) | morphogenesis | 304 | 2.41% |
| [GO:0030154](http://amigo.geneontology.org/cgi-bin/amigo/go.cgi?action=query&view=details&search_constraint=terms&query=GO:0030154) | cell differentiation | 215 | 1.71% |
| [GO:0006996](http://amigo.geneontology.org/cgi-bin/amigo/go.cgi?action=query&view=details&search_constraint=terms&query=GO:0006996) | organelle organization and biogenesis | 214 | 1.70% |
| [GO:0006950](http://amigo.geneontology.org/cgi-bin/amigo/go.cgi?action=query&view=details&search_constraint=terms&query=GO:0006950) | response to stress | 213 | 1.69% |
| [GO:0006412](http://amigo.geneontology.org/cgi-bin/amigo/go.cgi?action=query&view=details&search_constraint=terms&query=GO:0006412) | protein biosynthesis | 210 | 1.67% |
| [GO:0015031](http://amigo.geneontology.org/cgi-bin/amigo/go.cgi?action=query&view=details&search_constraint=terms&query=GO:0015031) | protein transport | 193 | 1.53% |
| [GO:0005975](http://amigo.geneontology.org/cgi-bin/amigo/go.cgi?action=query&view=details&search_constraint=terms&query=GO:0005975) | carbohydrate metabolism | 192 | 1.52% |
| [GO:0006629](http://amigo.geneontology.org/cgi-bin/amigo/go.cgi?action=query&view=details&search_constraint=terms&query=GO:0006629) | lipid metabolism | 173 | 1.37% |
| [GO:0009790](http://amigo.geneontology.org/cgi-bin/amigo/go.cgi?action=query&view=details&search_constraint=terms&query=GO:0009790) | embryonic development | 155 | 1.23% |
| [GO:0006811](http://amigo.geneontology.org/cgi-bin/amigo/go.cgi?action=query&view=details&search_constraint=terms&query=GO:0006811) | ion transport | 154 | 1.22% |
| [GO:0006519](http://amigo.geneontology.org/cgi-bin/amigo/go.cgi?action=query&view=details&search_constraint=terms&query=GO:0006519) | amino acid and derivative metabolism | 129 | 1.02% |
| [GO:0007049](http://amigo.geneontology.org/cgi-bin/amigo/go.cgi?action=query&view=details&search_constraint=terms&query=GO:0007049) | cell cycle | 127 | 1.01% |
| [GO:0006091](http://amigo.geneontology.org/cgi-bin/amigo/go.cgi?action=query&view=details&search_constraint=terms&query=GO:0006091) | generation of precursor metabolites and energy | 124 | 0.98% |
| [GO:0016265](http://amigo.geneontology.org/cgi-bin/amigo/go.cgi?action=query&view=details&search_constraint=terms&query=GO:0016265) | death | 110 | 0.87% |
| [GO:0008219](http://amigo.geneontology.org/cgi-bin/amigo/go.cgi?action=query&view=details&search_constraint=terms&query=GO:0008219) | cell death | 110 | 0.87% |
| [GO:0006259](http://amigo.geneontology.org/cgi-bin/amigo/go.cgi?action=query&view=details&search_constraint=terms&query=GO:0006259) | DNA metabolism | 106 | 0.84% |
| [GO:0007010](http://amigo.geneontology.org/cgi-bin/amigo/go.cgi?action=query&view=details&search_constraint=terms&query=GO:0007010) | cytoskeleton organization and biogenesis | 76 | 0.60% |
| [GO:0019725](http://amigo.geneontology.org/cgi-bin/amigo/go.cgi?action=query&view=details&search_constraint=terms&query=GO:0019725) | cell homeostasis | 67 | 0.53% |
| [GO:0009605](http://amigo.geneontology.org/cgi-bin/amigo/go.cgi?action=query&view=details&search_constraint=terms&query=GO:0009605) | response to external stimulus | 65 | 0.52% |
| [GO:0008283](http://amigo.geneontology.org/cgi-bin/amigo/go.cgi?action=query&view=details&search_constraint=terms&query=GO:0008283) | cell proliferation | 60 | 0.48% |
| [GO:0007267](http://amigo.geneontology.org/cgi-bin/amigo/go.cgi?action=query&view=details&search_constraint=terms&query=GO:0007267) | cell-cell signaling | 40 | 0.32% |
| [GO:0000003](http://amigo.geneontology.org/cgi-bin/amigo/go.cgi?action=query&view=details&search_constraint=terms&query=GO:0000003) | reproduction | 39 | 0.31% |
| [GO:0040007](http://amigo.geneontology.org/cgi-bin/amigo/go.cgi?action=query&view=details&search_constraint=terms&query=GO:0040007) | growth | 39 | 0.31% |
| [GO:0009607](http://amigo.geneontology.org/cgi-bin/amigo/go.cgi?action=query&view=details&search_constraint=terms&query=GO:0009607) | response to biotic stimulus | 34 | 0.27% |
| [GO:0009628](http://amigo.geneontology.org/cgi-bin/amigo/go.cgi?action=query&view=details&search_constraint=terms&query=GO:0009628) | response to abiotic stimulus | 32 | 0.25% |
| [GO:0007610](http://amigo.geneontology.org/cgi-bin/amigo/go.cgi?action=query&view=details&search_constraint=terms&query=GO:0007610) | behavior | 27 | 0.21% |
| [GO:0009719](http://amigo.geneontology.org/cgi-bin/amigo/go.cgi?action=query&view=details&search_constraint=terms&query=GO:0009719) | response to endogenous stimulus | 23 | 0.18% |
| [GO:0016049](http://amigo.geneontology.org/cgi-bin/amigo/go.cgi?action=query&view=details&search_constraint=terms&query=GO:0016049) | cell growth | 17 | 0.13% |
| [GO:0007005](http://amigo.geneontology.org/cgi-bin/amigo/go.cgi?action=query&view=details&search_constraint=terms&query=GO:0007005) | mitochondrion organization and biogenesis | 13 | 0.10% |
| [GO:0040029](http://amigo.geneontology.org/cgi-bin/amigo/go.cgi?action=query&view=details&search_constraint=terms&query=GO:0040029) | regulation of gene expression, epigenetic | 11 | 0.09% |
| [GO:0016032](http://amigo.geneontology.org/cgi-bin/amigo/go.cgi?action=query&view=details&search_constraint=terms&query=GO:0016032) | viral life cycle | 8 | 0.06% |
| [GO:0019748](http://amigo.geneontology.org/cgi-bin/amigo/go.cgi?action=query&view=details&search_constraint=terms&query=GO:0019748) | secondary metabolism | 5 | 0.04% |
| [GO:0008037](http://amigo.geneontology.org/cgi-bin/amigo/go.cgi?action=query&view=details&search_constraint=terms&query=GO:0008037) | cell recognition | 3 | 0.02% |
| [GO:0007028](http://amigo.geneontology.org/cgi-bin/amigo/go.cgi?action=query&view=details&search_constraint=terms&query=GO:0007028) | cytoplasm organization and biogenesis | 1 | 0.01% |

**CELLULAR COMPONENT GO terms**

| [**GO:0005575**](http://amigo.geneontology.org/cgi-bin/amigo/go.cgi?action=query&view=details&search_constraint=terms&query=GO:0005575) **Cellular component** | | | |
| --- | --- | --- | --- |
| **GO Class ID** | **Definitions** | **Counts** | **Fraction** |
| [GO:0005623](http://amigo.geneontology.org/cgi-bin/amigo/go.cgi?action=query&view=details&search_constraint=terms&query=GO:0005623) | cell | 3949 | 36.44% |
| [GO:0005622](http://amigo.geneontology.org/cgi-bin/amigo/go.cgi?action=query&view=details&search_constraint=terms&query=GO:0005622) | intracellular | 2729 | 25.18% |
| [GO:0005737](http://amigo.geneontology.org/cgi-bin/amigo/go.cgi?action=query&view=details&search_constraint=terms&query=GO:0005737) | cytoplasm | 1500 | 13.84% |
| [GO:0005634](http://amigo.geneontology.org/cgi-bin/amigo/go.cgi?action=query&view=details&search_constraint=terms&query=GO:0005634) | nucleus | 658 | 6.07% |
| [GO:0005886](http://amigo.geneontology.org/cgi-bin/amigo/go.cgi?action=query&view=details&search_constraint=terms&query=GO:0005886) | plasma membrane | 271 | 2.50% |
| [GO:0005739](http://amigo.geneontology.org/cgi-bin/amigo/go.cgi?action=query&view=details&search_constraint=terms&query=GO:0005739) | mitochondrion | 266 | 2.45% |
| [GO:0005856](http://amigo.geneontology.org/cgi-bin/amigo/go.cgi?action=query&view=details&search_constraint=terms&query=GO:0005856) | cytoskeleton | 230 | 2.12% |
| [GO:0005576](http://amigo.geneontology.org/cgi-bin/amigo/go.cgi?action=query&view=details&search_constraint=terms&query=GO:0005576) | extracellular region | 214 | 1.97% |
| [GO:0005783](http://amigo.geneontology.org/cgi-bin/amigo/go.cgi?action=query&view=details&search_constraint=terms&query=GO:0005783) | endoplasmic reticulum | 161 | 1.49% |
| [GO:0005829](http://amigo.geneontology.org/cgi-bin/amigo/go.cgi?action=query&view=details&search_constraint=terms&query=GO:0005829) | cytosol | 150 | 1.38% |
| [GO:0005840](http://amigo.geneontology.org/cgi-bin/amigo/go.cgi?action=query&view=details&search_constraint=terms&query=GO:0005840) | ribosome | 149 | 1.37% |
| [GO:0005794](http://amigo.geneontology.org/cgi-bin/amigo/go.cgi?action=query&view=details&search_constraint=terms&query=GO:0005794) | Golgi apparatus | 137 | 1.26% |
| [GO:0005694](http://amigo.geneontology.org/cgi-bin/amigo/go.cgi?action=query&view=details&search_constraint=terms&query=GO:0005694) | chromosome | 67 | 0.62% |
| [GO:0005654](http://amigo.geneontology.org/cgi-bin/amigo/go.cgi?action=query&view=details&search_constraint=terms&query=GO:0005654) | nucleoplasm | 60 | 0.55% |
| [GO:0005768](http://amigo.geneontology.org/cgi-bin/amigo/go.cgi?action=query&view=details&search_constraint=terms&query=GO:0005768) | endosome | 44 | 0.41% |
| [GO:0016023](http://amigo.geneontology.org/cgi-bin/amigo/go.cgi?action=query&view=details&search_constraint=terms&query=GO:0016023) | cytoplasmic membrane-bound vesicle | 43 | 0.40% |
| [GO:0005615](http://amigo.geneontology.org/cgi-bin/amigo/go.cgi?action=query&view=details&search_constraint=terms&query=GO:0005615) | extracellular space | 37 | 0.34% |
| [GO:0005730](http://amigo.geneontology.org/cgi-bin/amigo/go.cgi?action=query&view=details&search_constraint=terms&query=GO:0005730) | nucleolus | 28 | 0.26% |
| [GO:0005773](http://amigo.geneontology.org/cgi-bin/amigo/go.cgi?action=query&view=details&search_constraint=terms&query=GO:0005773) | vacuole | 26 | 0.24% |
| [GO:0005578](http://amigo.geneontology.org/cgi-bin/amigo/go.cgi?action=query&view=details&search_constraint=terms&query=GO:0005578) | extracellular matrix (sensu Metazoa) | 24 | 0.22% |
| [GO:0005764](http://amigo.geneontology.org/cgi-bin/amigo/go.cgi?action=query&view=details&search_constraint=terms&query=GO:0005764) | lysosome | 23 | 0.21% |
| [GO:0005815](http://amigo.geneontology.org/cgi-bin/amigo/go.cgi?action=query&view=details&search_constraint=terms&query=GO:0005815) | microtubule organizing center | 22 | 0.20% |
| [GO:0005635](http://amigo.geneontology.org/cgi-bin/amigo/go.cgi?action=query&view=details&search_constraint=terms&query=GO:0005635) | nuclear membrane | 17 | 0.16% |
| [GO:0005777](http://amigo.geneontology.org/cgi-bin/amigo/go.cgi?action=query&view=details&search_constraint=terms&query=GO:0005777) | peroxisome | 12 | 0.11% |
| [GO:0000228](http://amigo.geneontology.org/cgi-bin/amigo/go.cgi?action=query&view=details&search_constraint=terms&query=GO:0000228) | nuclear chromosome | 12 | 0.11% |
| [GO:0005929](http://amigo.geneontology.org/cgi-bin/amigo/go.cgi?action=query&view=details&search_constraint=terms&query=GO:0005929) | cilium | 5 | 0.05% |
| [GO:0005811](http://amigo.geneontology.org/cgi-bin/amigo/go.cgi?action=query&view=details&search_constraint=terms&query=GO:0005811) | lipid particle | 3 | 0.03% |

**MOLECULAR FUNCTION GO terms**

| [**GO:0003674**](http://amigo.geneontology.org/cgi-bin/amigo/go.cgi?action=query&view=details&search_constraint=terms&query=GO:0003674) **Molecular function** | | | |
| --- | --- | --- | --- |
| **GO Class ID** | **Definitions** | **Counts** | **Fraction** |
| [GO:0005488](http://amigo.geneontology.org/cgi-bin/amigo/go.cgi?action=query&view=details&search_constraint=terms&query=GO:0005488) | binding | 3795 | 28.52% |
| [GO:0003824](http://amigo.geneontology.org/cgi-bin/amigo/go.cgi?action=query&view=details&search_constraint=terms&query=GO:0003824) | catalytic activity | 2156 | 16.20% |
| [GO:0005515](http://amigo.geneontology.org/cgi-bin/amigo/go.cgi?action=query&view=details&search_constraint=terms&query=GO:0005515) | protein binding | 1160 | 8.72% |
| [GO:0016787](http://amigo.geneontology.org/cgi-bin/amigo/go.cgi?action=query&view=details&search_constraint=terms&query=GO:0016787) | hydrolase activity | 781 | 5.87% |
| [GO:0003676](http://amigo.geneontology.org/cgi-bin/amigo/go.cgi?action=query&view=details&search_constraint=terms&query=GO:0003676) | nucleic acid binding | 665 | 5.00% |
| [GO:0000166](http://amigo.geneontology.org/cgi-bin/amigo/go.cgi?action=query&view=details&search_constraint=terms&query=GO:0000166) | nucleotide binding | 657 | 4.94% |
| [GO:0016740](http://amigo.geneontology.org/cgi-bin/amigo/go.cgi?action=query&view=details&search_constraint=terms&query=GO:0016740) | transferase activity | 643 | 4.83% |
| [GO:0003677](http://amigo.geneontology.org/cgi-bin/amigo/go.cgi?action=query&view=details&search_constraint=terms&query=GO:0003677) | DNA binding | 365 | 2.74% |
| [GO:0005215](http://amigo.geneontology.org/cgi-bin/amigo/go.cgi?action=query&view=details&search_constraint=terms&query=GO:0005215) | transporter activity | 304 | 2.28% |
| [GO:0016301](http://amigo.geneontology.org/cgi-bin/amigo/go.cgi?action=query&view=details&search_constraint=terms&query=GO:0016301) | kinase activity | 282 | 2.12% |
| [GO:0004871](http://amigo.geneontology.org/cgi-bin/amigo/go.cgi?action=query&view=details&search_constraint=terms&query=GO:0004871) | signal transducer activity | 254 | 1.91% |
| [GO:0008233](http://amigo.geneontology.org/cgi-bin/amigo/go.cgi?action=query&view=details&search_constraint=terms&query=GO:0008233) | peptidase activity | 222 | 1.67% |
| [GO:0030528](http://amigo.geneontology.org/cgi-bin/amigo/go.cgi?action=query&view=details&search_constraint=terms&query=GO:0030528) | transcription regulator activity | 198 | 1.49% |
| [GO:0004872](http://amigo.geneontology.org/cgi-bin/amigo/go.cgi?action=query&view=details&search_constraint=terms&query=GO:0004872) | receptor activity | 192 | 1.44% |
| [GO:0005198](http://amigo.geneontology.org/cgi-bin/amigo/go.cgi?action=query&view=details&search_constraint=terms&query=GO:0005198) | structural molecule activity | 185 | 1.39% |
| [GO:0004672](http://amigo.geneontology.org/cgi-bin/amigo/go.cgi?action=query&view=details&search_constraint=terms&query=GO:0004672) | protein kinase activity | 180 | 1.35% |
| [GO:0003723](http://amigo.geneontology.org/cgi-bin/amigo/go.cgi?action=query&view=details&search_constraint=terms&query=GO:0003723) | RNA binding | 145 | 1.09% |
| [GO:0008289](http://amigo.geneontology.org/cgi-bin/amigo/go.cgi?action=query&view=details&search_constraint=terms&query=GO:0008289) | lipid binding | 142 | 1.07% |
| [GO:0005509](http://amigo.geneontology.org/cgi-bin/amigo/go.cgi?action=query&view=details&search_constraint=terms&query=GO:0005509) | calcium ion binding | 123 | 0.92% |
| [GO:0030234](http://amigo.geneontology.org/cgi-bin/amigo/go.cgi?action=query&view=details&search_constraint=terms&query=GO:0030234) | enzyme regulator activity | 116 | 0.87% |
| [GO:0005102](http://amigo.geneontology.org/cgi-bin/amigo/go.cgi?action=query&view=details&search_constraint=terms&query=GO:0005102) | receptor binding | 113 | 0.85% |
| [GO:0003700](http://amigo.geneontology.org/cgi-bin/amigo/go.cgi?action=query&view=details&search_constraint=terms&query=GO:0003700) | transcription factor activity | 97 | 0.73% |
| [GO:0008092](http://amigo.geneontology.org/cgi-bin/amigo/go.cgi?action=query&view=details&search_constraint=terms&query=GO:0008092) | cytoskeletal protein binding | 94 | 0.71% |
| [GO:0045182](http://amigo.geneontology.org/cgi-bin/amigo/go.cgi?action=query&view=details&search_constraint=terms&query=GO:0045182) | translation regulator activity | 63 | 0.47% |
| [GO:0008135](http://amigo.geneontology.org/cgi-bin/amigo/go.cgi?action=query&view=details&search_constraint=terms&query=GO:0008135) | translation factor activity, nucleic acid binding | 61 | 0.46% |
| [GO:0004721](http://amigo.geneontology.org/cgi-bin/amigo/go.cgi?action=query&view=details&search_constraint=terms&query=GO:0004721) | phosphoprotein phosphatase activity | 59 | 0.44% |
| [GO:0003779](http://amigo.geneontology.org/cgi-bin/amigo/go.cgi?action=query&view=details&search_constraint=terms&query=GO:0003779) | actin binding | 57 | 0.43% |
| [GO:0005216](http://amigo.geneontology.org/cgi-bin/amigo/go.cgi?action=query&view=details&search_constraint=terms&query=GO:0005216) | ion channel activity | 52 | 0.39% |
| [GO:0004518](http://amigo.geneontology.org/cgi-bin/amigo/go.cgi?action=query&view=details&search_constraint=terms&query=GO:0004518) | nuclease activity | 38 | 0.29% |
| [GO:0003774](http://amigo.geneontology.org/cgi-bin/amigo/go.cgi?action=query&view=details&search_constraint=terms&query=GO:0003774) | motor activity | 29 | 0.22% |
| [GO:0030246](http://amigo.geneontology.org/cgi-bin/amigo/go.cgi?action=query&view=details&search_constraint=terms&query=GO:0030246) | carbohydrate binding | 26 | 0.20% |
| [GO:0003682](http://amigo.geneontology.org/cgi-bin/amigo/go.cgi?action=query&view=details&search_constraint=terms&query=GO:0003682) | chromatin binding | 21 | 0.16% |
| [GO:0016209](http://amigo.geneontology.org/cgi-bin/amigo/go.cgi?action=query&view=details&search_constraint=terms&query=GO:0016209) | antioxidant activity | 17 | 0.13% |
| [GO:0019825](http://amigo.geneontology.org/cgi-bin/amigo/go.cgi?action=query&view=details&search_constraint=terms&query=GO:0019825) | oxygen binding | 7 | 0.05% |
| [GO:0005326](http://amigo.geneontology.org/cgi-bin/amigo/go.cgi?action=query&view=details&search_constraint=terms&query=GO:0005326) | neurotransmitter transporter activity | 6 | 0.05% |
