# Supplementary material for: Development of an oligo DNA microarray for the European sea bass and its application to expression profiling of jaw deformity
Source: BMC Genomics. 2010 Jun 3;11:354. doi: 10.1186/1471-2164-11-354 (PMC2889902; doi:10.1186/1471-2164-11-354)
Supplement: Additional file 2 — Microarray quality assessment. Hybridization success across all experiments (Figure S1) and distribution of observed fold changes between Probe_1 and Probe_2 (Figure S2) are reported. [file 1471-2164-11-354-S2.PDF]

Microarray quality assessment

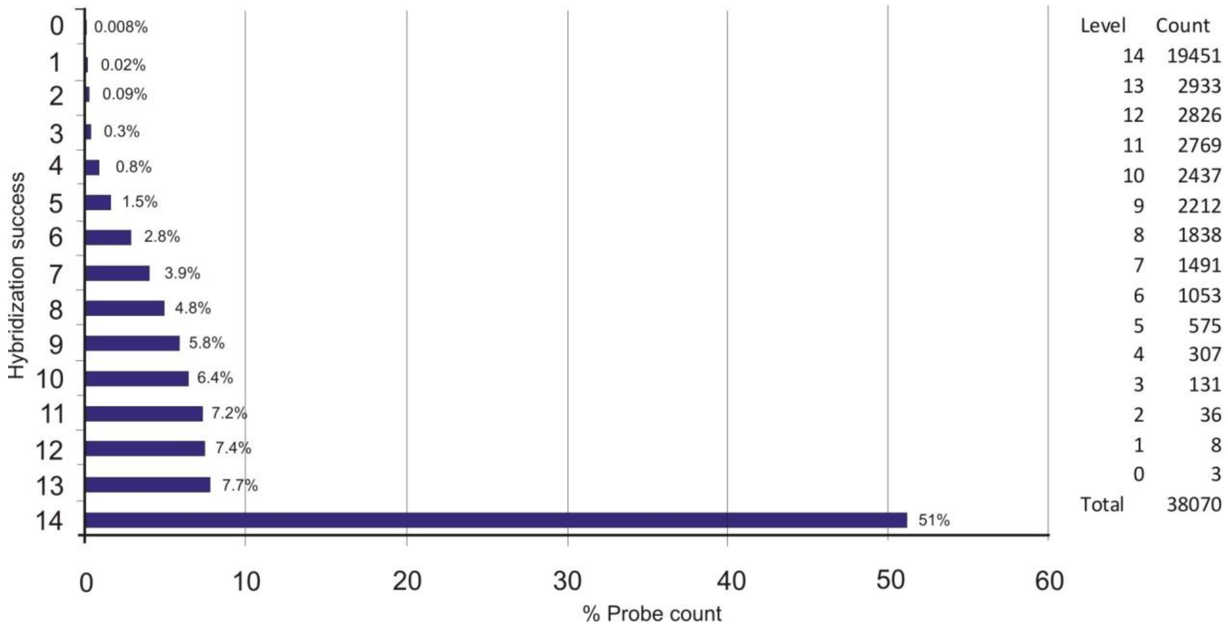

Figure S1. Distribution analysis of hybridization success across 14 microarray experiments.
For each probe, the number of times it was called "present" by Agilent *Feature Extraction 9.5.1* software was calculated. On the y-axis, the number of positive calls in 14 experiments (0 corresponds to probes that never hybridized, 14 corresponds to probes that always successfully hybridized). On the x-axis is the number of probes falling into each group (0-14). The exact count of probes and the corresponding percentage are also reported for each group.

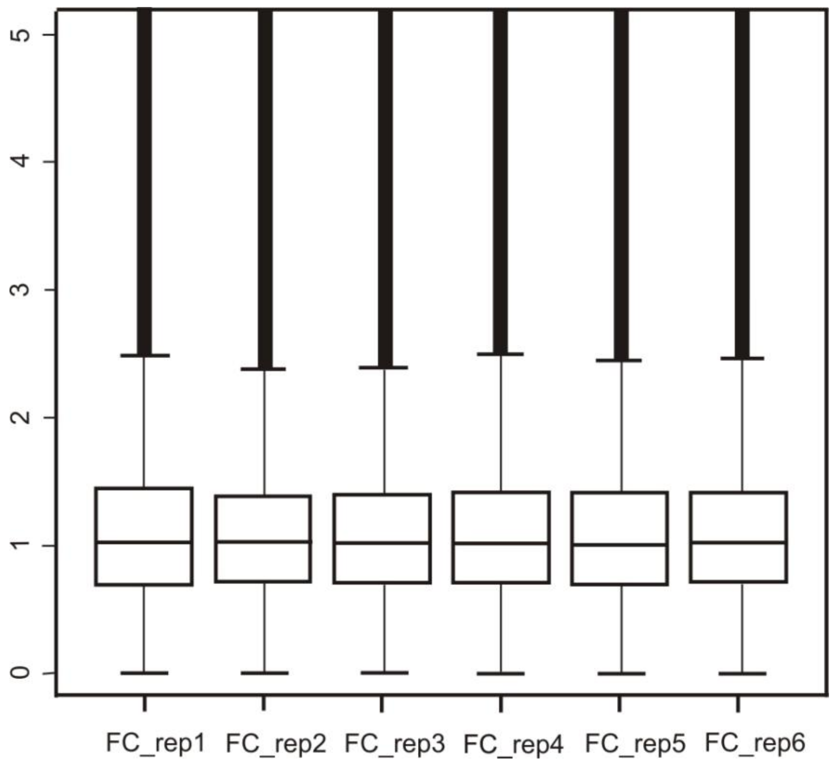

Figure S2. Correlation between levels of gene expression measured by Probe_1 and Probe_2.
Each plot describes the distribution of observed fold-changes between Probe_1 and Probe_2 for each array experiment in 38 days-old sea bass heads. On the y-axis are the Fold-change (expression values ratio between Probe_1 and Probe_2) distributions calculated for each biological replicate of Stage 38 (x-axis).
