# Supplementary figures and images for: Development of an oligo DNA microarray for the European sea bass and its application to expression profiling of jaw deformity
Source: BMC Genomics. 2010 Jun 3;11:354. doi: 10.1186/1471-2164-11-354 (PMC2889902; doi:10.1186/1471-2164-11-354)

# DLPD

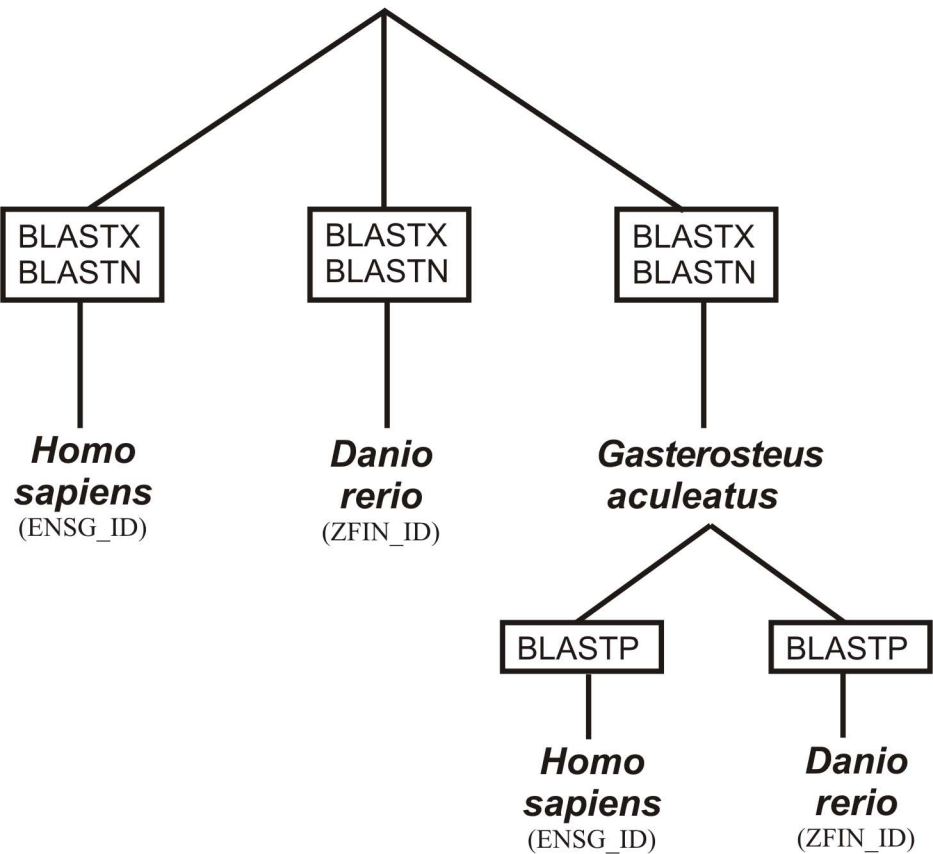

Supplement: Additional file 4 — Scheme illustrating the four different approaches tested for retrieving DAVID identifiers. DLPD entries were linked either to human Ensembl Gene IDs or to zebrafish ZFIN IDs directly or passing through stickleback sequences. [file 1471-2164-11-354-S4.PDF]
