# Supplementary material for: Development of an oligo DNA microarray for the European sea bass and its application to expression profiling of jaw deformity
Source: BMC Genomics. 2010 Jun 3;11:354. doi: 10.1186/1471-2164-11-354 (PMC2889902; doi:10.1186/1471-2164-11-354)
Supplement: Additional file 5 — PANTHER terms significantly represented among differentially expressed genes. Results obtained using DAVID (Database for Annotation, Visualization, and Integrated Discovery) 2008. [file 1471-2164-11-354-S5.DOC]

| **Category** | **Term** | **Count** | **%** | ***p-value*** | **Fold Enrichment** | **FDR** |
| --- | --- | --- | --- | --- | --- | --- |
| **PANTHER_BP_ALL** | BP00114:MAPKKK cascade | 5 | 8.33% | 0.00859 | 6.04 | 10.38 |
|  | BP00112:Calcium mediated signaling | 5 | 8.33% | 0.04280208016815121 | 3.7 | 42.6 |
|  | BP00182:Sensory perception | 8 | 13.33% | 0.0018541833370723524 | 4.4 | 2.3 |
|  | BP00046:Other mRNA transcription | 5 | 8.33% | 0.0226930832535855 | 4.5 | 25.2 |
|  | BP00193:Developmental processes | 13 | 21.67% | 0.0044420590821753975 | 2.4 | 5.5 |
| **PANTHER_MF_ALL** | MF00257:CREB transcription factor | 4 | 6.67% | 0.005133714219021819 | 11 | 6.4 |
|  | MF00017:Cytokine | 4 | 6.67% | 0.08986335581108551 | 3.7 | 70.2 |
|  | MF00016:Signaling molecule | 6 | 10.00% | 0.06905802859370685 | 2.7 | 60.1 |
|  | MF00128:Oxidase | 4 | 6.67% | 0.0878466745835501 | 3.7 | 69.3 |
|  | MF00091:Cytoskeletal protein | 9 | 15.00% | 0.005942642286868892 | 3.1 | 7.4 |
|  | MF00022:Neurotrophic factor | 4 | 6.67% | 0.09603006061804444 | 3.6 | 72.7 |

PANTHER terms significantly represented among differentially expressed genes

| **Category** | **Term** | **Count** | **%** | ***p-value*** | **Fold Enrichment** | **FDR** |
| --- | --- | --- | --- | --- | --- | --- |
| **GOTERM_BP_ALL** | GO:0050877~neurological system process | 8 | 13.33% | 0.006574 | 3.468007 | 11.51896 |
|  | GO:0048856~anatomical structure development | 14 | 23.33% | 0.026081 | 1.852929 | 38.75987 |
|  | GO:0003008~system process | 9 | 15.00% | 0.022777 | 2.484961 | 34.78694 |
|  | GO:0032501~multicellular organismal process | 23 | 38.33% | 2.19E-04 | 2.116818 | 0.406379 |
|  | GO:0050789~regulation of biological process | 24 | 40.00% | 0.01166 | 1.561735 | 19.5573 |
|  | GO:0007154~cell communication | 19 | 31.67% | 0.034946 | 1.558464 | 48.31694 |
|  | GO:0007399~nervous system development | 7 | 11.67% | 0.039474 | 2.695892 | 52.63508 |
|  | GO:0007275~multicellular organismal development | 15 | 25.00% | 0.011784 | 1.970747 | 19.74412 |
|  | GO:0007268~synaptic transmission | 6 | 10.00% | 0.002618 | 6.04953 | 4.747339 |
|  | GO:0006811~ion transport | 7 | 11.67% | 0.022343 | 3.079132 | 34.24804 |
|  | GO:0048731~system development | 13 | 21.67% | 0.013046 | 2.117152 | 21.62432 |
|  | GO:0001505~regulation of neurotransmitter levels | 4 | 6.67% | 0.002972 | 13.29403 | 5.3725 |
|  | GO:0019226~transmission of nerve impulse | 6 | 10.00% | 0.005133 | 5.177002 | 9.107265 |
|  | GO:0065007~biological regulation | 26 | 43.33% | 0.011622 | 1.508146 | 19.49988 |
| **GOTERM_CC_ALL** | GO:0005856~cytoskeleton | 10 | 16.67% | 0.013466 | 2.513273 | 18.45919 |
| **GOTERM_MF_ALL** | GO:0008092~cytoskeletal protein binding | 6 | 10.00% | 0.046655 | 2.96898 | 56.07969 |
| **UP_TISSUE** | Brain | 39 | 65.00% | 1.78E-04 | 1.584613 | 0.254897 |
