# Supplementary material for: Development of an oligo DNA microarray for the European sea bass and its application to expression profiling of jaw deformity
Source: BMC Genomics. 2010 Jun 3;11:354. doi: 10.1186/1471-2164-11-354 (PMC2889902; doi:10.1186/1471-2164-11-354)

A. Sense overexpressed

PROBE	*G.aculeatus* homologues	E-value	Subject orientation	Fluorescence signal	FC
**DLPD03928**	ENSGACT000000021382	5.00^{-30}	+1	148857	2589
**DLPD06020**		1.00^{-177}	-1	58	

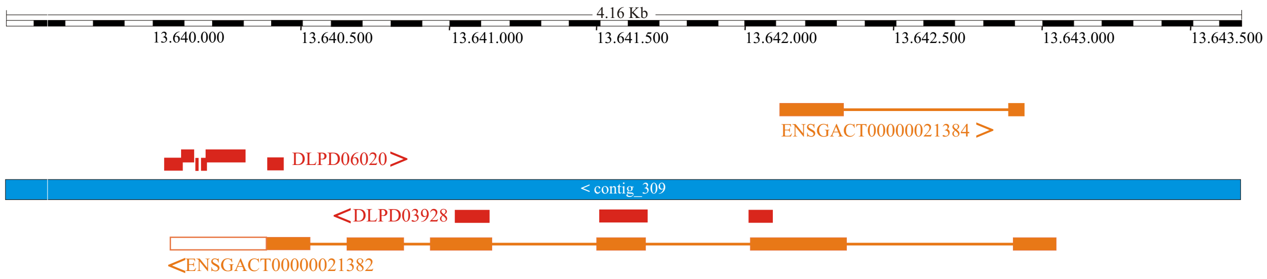

B. Antisense overexpressed

PROBE	*G.aculeatus* homologues	E-value	Subject orientation	Fluorescence signal	FC
**DLPD10744**	ENSGACT00000007663	1.00^{-39}	+1	7	0.016
**DLPD01599**		3.00^{-36}	-1	441	

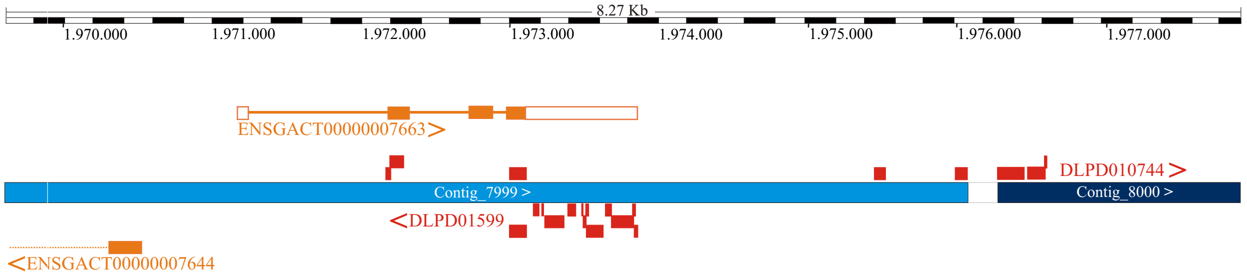

Supplement: Additional file 6 — Examples of DLPD entries matching, on opposite orientation, the same stickleback transcripts. A. DLPD03928 shares the same orientation with ENGACT000000021382 and shows higher levels of expression compared to the corresponding antisense transcript (DLPD06020). The opposite situation is represented in B. DLPD01599 has opposite orientation to its putative stickleback homologue (ENGACT000000021382) and is over-expressed compared to the corresponding sense transcripts (DLPD10744). Blast E-value, microarray fluorescence signal for each DLPD entry, and fold change between sense and antisense DLPD transcripts are also reported. [file 1471-2164-11-354-S6.PDF]
